# Comparing dosimetric performance of robust and planning target volume‐based optimization in photon lung stereotactic radiotherapy

**DOI:** 10.1002/mp.70219

**Published:** 2025-12-29

**Authors:** Maude Gondré, Véronique Vallet, Fernanda Herrera, Raphael Moeckli

**Affiliations:** ^1^ Institute of Radiation Physics Lausanne University Hospital and Lausanne University Lausanne Switzerland; ^2^ Radio‐Oncology Department Lausanne University Hospital and Lausanne University Lausanne Switzerland

**Keywords:** CyberKnife, lung SBRT, PTV‐based planning, Robust optimization, Synchrony tracking, X‐sight spine tracking

## Abstract

**Background:**

Stereotactic body radiation therapy (SBRT) is an effective treatment for lung tumors but it is challenged by respiratory motion and setup uncertainties. These are traditionally managed by optimizing on a planning target volume (PTV). Robust optimization offers an alternative by accounting for uncertainties through error scenarios. Although well established in proton therapy, its application in photon‐based lung SBRT has mostly been limited to setup uncertainties and internal target volume (ITV)‐based planning. This study is the first to evaluate robust optimization applied directly to the gross tumor volume (GTV) in combination with tracking modalities of the CyberKnife installation.

**Purpose:**

This study evaluates the benefit of using robust optimization combined with CyberKnife tracking modalities on ipsilateral lung dose in lung SBRT.

**Methods:**

CyberKnife (Accuray Inc., USA) lung plans were optimized using both PTV‐based and robust approaches. Two tracking modalities were evaluated: Synchrony (real‐time respiratory motion compensation) and x‐sight spine (static tracking). The robustness of the plans was evaluated by simulating setup uncertainties and tumor motion using 4D‐CT. GTV coverage and lung dose metrics—V20Gy, V5Gy, mean lung dose (MLD)—were compared across uncertainty scenarios.

**Results:**

Both planning methods achieved more than 99.5% of scenarios meeting the GTV coverage constraint for both tracking modalities. Robust optimization reduced lung dose. The V20Gy was reduced from 4.5% to 3.2% for Synchrony and from 9.3% to 6.9% for x‐sight spine. The V5Gy decreased from 22.3% to 18.0% for Synchrony and from 35.0% to 30.2% for x‐sight spine. The MLD was reduced with robust optimization by 0.6 Gy for Synchrony and 1.3 Gy for x‐sight spine.

**Conclusions:**

This study demonstrates the benefit of combining robust optimization with tracking techniques in photon lung SBRT for reducing lung dose and introduces a novel treatment planning strategy with the potential to improve both dosimetric outcomes and clinical consistency.

## 1 INTRODUCTION

Lung cancer is the leading cause of cancer‐related deaths worldwide, as reported by the World Health Organization.^1^ Stereotactic body radiotherapy (SBRT), combining high dose of radiation delivered in only few fractions, has proved to be an effective treatment option.^2^, ^3^ A common side effect of lung SBRT is the development of radiation pneumonitis, the severity of which is correlated with the lung volume receiving 20 Gy (V20Gy), 5 Gy (V5Gy) and the mean lung dose (MLD).^4^, ^5^, ^6^ One of the major factors influencing both tumor coverage and normal tissue toxicity in lung SBRT is respiratory motion, which can significantly compromise the accuracy of dose delivery. This issue is typically addressed using 4D‐CT to assess the tumor position throughout the respiratory cycle. The gross tumor volume (GTV) is delineated on each phase of the 4D‐CT, and the union of these GTVs defines the internal target volume (ITV),^7^ ensuring adequate tumor coverage despite respiratory motion. To compensate for setup uncertainties, an additional margin is added to the ITV to create the planning target volume (PTV). The CyberKnife (Accuray Inc., USA) system has gone a step further by enabling real‐time tumor tracking during treatment delivery.^8^ In this approach, the margin for setup uncertainty is applied directly to the GTV, with no need to create an ITV, thus limiting the amount of healthy tissue irradiated.

Another challenge in lung radiotherapy arises from the density difference between the lung (low density) and the tumor (high density). Indeed, the PTV is created based on the high‐density tumor, to which a safety margin—composed solely of low‐density lung tissue—is added, resulting in a heterogeneous PTV. During treatment delivery, the tumor may be located at any point inside the PTV, leading to regions of high‐density tissue being irradiated with fluence optimized for low‐density tissue, and vice versa. This discrepancy can significantly impact the accuracy of dose delivery.^9^, ^10^ Several strategies have been proposed to mitigate this issue. One method involves overriding the density of the PTV to 1 g/cm^3^ during dose calculation.^11^ Another approach was proposed using the combination of type A and type B dose calculation algorithms.^12^ This method optimizes the fluence using the type‐A algorithm, which does not account for changes in electron transport, and subsequently recalculates the final dose with the type B algorithm.^9^

A promising solution to both the challenges outlined above is robust optimization.^13^ This method accounts for uncertainties—such as patient setup errors and organ motion—by including predefined error scenarios into the optimization process. The objective function is minimized across all these scenarios, allowing uncertainties to be directly addressed during optimization. This approach eliminates the need for a PTV and instead, optimizes directly on the GTV (in GTV‐based plans), or the ITV (in ITV‐based plans), depending on the type of uncertainty considered. Additionally, by optimizing directly on the high‐density GTV, robust optimization avoids the dosimetric inconsistencies introduced by the heterogeneous PTV.

Robust optimization is well established in proton therapy, but its application in photon therapy has been less common. In recent years, however, this technique has gained popularity in photon irradiation, particularly for lung SBRT.^14^, ^15^, ^16^, ^17^ Despite this, robust optimization for photon‐based lung SBRT has typically been applied to the ITV, primarily addressing setup uncertainties. In this study, we evaluate robust optimization applied directly to the GTV, combined with two CyberKnife tracking modalities: Synchrony, which enables real‐time respiratory motion compensation, and x‐sight spine, a static tracking method. To the best of our knowledge, this is the first study to investigate the benefit of GTV‐based robust optimization combined with tracking techniques on ipsilateral lung dose compared to conventional PTV‐based planning.

## 2 MATERIALS AND METHODS

### 2.1 CyberKnife tracking modalities and volumes definition

#### 2.1.1 Synchrony tracking modality

The Synchrony tracking method allows real‐time tracking of the lung lesion according to the respiratory motion during the treatment delivery. To achieve this, a correlation model is established between the patient's breathing pattern, represented by LEDs positioned on the patient's thorax, and fiducials implanted in or near the lung lesion serving as surrogates for tumor position. As a result, the tumor's location during the respiratory cycle is continuously tracked, allowing the CyberKnife robot to dynamically adapt beam delivery accordingly. During treatment, periodic images update the correlation model.^18^, ^19^ If the tumor's actual position, as determined from the images, deviates beyond a predefined threshold from the model's predicated position, the beam stops, and a new model is created. Since this tracking modality accounts for respiratory motion in real time, there is no need to consider tumor movement during plan optimization. As a result, the GTV can be directly delineated on the expiratory phase CT, and an isotropic setup margin of 3 mm is added to create the PTV.

#### 2.1.2 X‐sight spine tracking modality

With the x‐sight spine tracking method, tracking is performed on skeletal structures in the spine, using image acquisitions prior and during treatment delivery. In the case of lung SBRT, tracking is performed on the vertebra closest to the lung lesion. Unlike treatments using the Synchrony system, this method does not allow real‐time tracking of the lung tumor during patient's breathing. Therefore, GTV motion due to breathing must be accounted for during the planning process. This is typically done by acquiring a 4D‐CT with 10 respiratory phases^20^, ^21^ in addition to the expirium phase. The ITV is defined as the union of GTVs delineated on each phase, and an isotropic 3 mm margin is added to create the PTV.

### 2.2 Treatment plans optimization

Treatment plans were optimized using the RayStation treatment planning system (TPS) version 12A (RaySearch Laboratories, Sweden),^22^, ^23^ which supports robust optimization based on the “minimax” method.^24^, ^25^ The study included 24 patients: 15 treated with Synchrony tracking and 9 with x‐sight spine tracking.

For the Synchrony tracking modality, three plans were optimized per patient:

‐ a PTV‐based plan, representing the traditional optimization approach in photon‐based lung SBRT
‐ a density override (DO) PTV‐based plan, where the PTV density was overridden to 1 g/cm^3^ to investigate the impact of approximating the PTV as homogeneous on the dose distribution
‐ a GTV‐based plan with robust optimization accounting for setup uncertainty

For x‐sight spine tracking modality, five plans were optimized per patient:

‐ a PTV‐based plan, the traditional planning approach
‐ a DO PTV‐based plan, with the PTV density overridden to 1 g/cm^3^
‐ an ITV‐based plan, with robust optimization accounting for setup uncertainty
‐ a DO ITV‐based plan, with the ITV overridden to 1 g/cm^3^, and with robust optimization for setup uncertainty
‐ a GTV‐based plan, with robust optimization considering both organ motion and setup uncertainties

Setup uncertainty for robust optimization was defined based on the setup margin used to create the PTV—from the GTV for Synchrony plans, or the ITV for x‐sight spine plans—resulting in seven error scenarios: one with no shift, and six with ± 3 mm shifts along the inferior–superior, anterior–posterior and right–left axes. For GTV‐based plans with x‐sight spine tracking modality, where organ motion is included in the robust optimization, these seven scenarios were applied to each phase of the 4D‐CT in addition to the expirium CT, resulting in 84 scenarios. Robust uncertainties were considered as systematic, meaning the same uncertainty was applied for all fractions.

The specifications of each plan are summarized in Table 1.

**TABLE 1:** Definition of plans for Synchrony and x‐sight spine tracking modalities with respect to setup and organ motion uncertainties, application of robustness (including number of error scenarios, if any), computational time relative to PTV‐based plan optimization (= for similar, + for moderate increase, and ++ for significant increase), and presence of inhomogeneity within the target volume.

Tracking modality	Optimization method	Setup uncertainty	Organ motion uncertainty	Robustness (no. of scenarios)	Computational time	Target volume inhomogeneity
Synchrony	PTV‐based	PTV = GTV + 3 mm	Real‐time racking	No	=	Yes
	DO PTV‐based	PTV = GTV + 3 mm	Real‐time racking	No	=	No (PTV density = 1 g/cm^3^)
	GTV‐based	3 mm robustness	Real‐time racking	Yes (7)	+	No
X‐sight spine	PTV‐based	PTV = ITV + 3 mm	Covered by ITV	No	=	Yes
	DO PTV‐based	PTV = ITV + 3 mm	Covered by ITV	No	=	No (PTV density = 1 g/cm^3^)
	ITV‐based	3 mm robustness	Covered by ITV	Yes (7)	+	Yes
	DO ITV‐based	3 mm robustness	Covered by ITV	Yes (7)	+	No (ITV density = 1 g/cm^3^)
	GTV‐based	3 mm robustness	4D‐CT robustness	Yes (84)	++	No

All patients received a prescription dose of 55 Gy delivered in 5 fractions, with a prescribed isodose of 80%. Plans were optimized using the multileaf collimator (MLC) and respected the dose constraints and objectives listed in Table S1.

Target dose objectives were a minimum dose of 55 Gy and a maximum dose of 68.75 Gy. These objectives were applied to the PTV for PTV‐ based plans, and to the ITV or GTV—defined as robust objective functions—for ITV‐ and GTV‐based plans, respectively. A robust objective function is minimized across all defined error scenarios.

To minimize dose to surrounding healthy tissues, dose fall‐off was optimized using a ring structure defined as (PTV+3cm)−PTV. Specific dose reductions were requested at 3 mm and 3 cm from the target volume. Given the typically small target volumes treated with the CyberKnife, and its inherent steep dose gradients, optimization on individual organs was generally unnecessary. However, for patients with a target volume in contact with the ribs—6 with Synchrony tracking and 3 with x‐sight spine tracking—an additional objective function was used to limit the maximum rib dose to below 43 Gy, as defined in Table S1. For ITV‐ and GTV‐based plans, the rib dose constraint was defined as robust.

### 2.3 Plan robustness evaluation

After optimization, the robustness of each plan was evaluated by simulating patient mispositioning and, for x‐sight spine plans, GTV motion through the respiratory cycle, and recomputing the dose distribution. The maximum simulated mispositioning was 3 mm in all directions, corresponding to the setup margin of PTV‐based plans, and to the setup uncertainty of ITV‐ and GTV‐based plans. 15 positional uncertainties, combining extremum and intermediate patient shifts were evaluated on the expirium CT for Synchrony patients, leading to a total of 15 simulating scenarios. For x‐sight spine patients, the same 15 positional uncertainties were applied to each phase of the 4D‐CT, in addition to the expirium CT, leading to 180 scenarios.

For all simulated scenarios of each plan type, several dosimetric parameters were evaluated, including the GTV coverage— defined as the volume of the GTV receiving at least 55 Gy (V55Gy)— the maximum dose to the GTV, and the ipsilateral lung dose, characterized by the volume receiving 20 Gy (V20Gy), 5 Gy (V5Gy) as well as the MLD. In addition, the percentage of simulated scenarios in which the GTV coverage exceeds 95% was recorded. A plan was considered robust if 95% of the GTV received the prescribed dose in all simulated scenarios. When applicable, the maximum dose to the rib, and the percentage of scenarios in which the rib constraint was met were also reported. The average values of the aforementioned dosimetric parameters were calculated across all simulated scenarios for each plan type and then, averaged over all patients, separately for the Synchrony and x‐sight spine tracking modalities. Corresponding 95% confidence intervals were calculated using Student's t‐distribution. For lung dose metrics (V20Gy, V5Gy, and MLD), *p*‐values were calculated using the Wilcoxon signed‐rank test, with the values obtained from the PTV‐based plan used as reference. A *p*‐value < 0.05 was considered statistically significant. No statistical comparison was performed for the rib due to insufficient data.

The correlation between both the PTV‐to‐GTV volume ratio and the GTV volume with the difference in V20Gy (ΔV20Gy), V5Gy (ΔV5Gy) and MLD (ΔMLD) between PTV‐based and GTV‐based plans was assessed using Spearman's rank correlation test for both tracking modalities. The corresponding *p*‐values were calculated, with a significance threshold set at 0.05. According to conventional interpretation, a Spearman correlation coefficient *r* below ± 0.3 indicates no or weak correlation, values between ± 0.3 and ± 0.7 indicate a moderate correlation, and values above ± 0.7 represent a strong correlation. The number of monitor units (MU) of each plan was also compared.

## 3 RESULTS

Table 2 presents the averaged results, with 95% confidence intervals in bracket, for each plan type for both Synchrony and x‐sight spine tracking modalities, including the percentage of scenarios where GTV coverage exceeded 95%, as well as the GTV coverage (V55Gy) and maximum GTV dose, and the dosimetric results for the ipsilateral lung (V20Gy, V5Gy, and MLD). The results are averaged over all simulated scenarios and patients for each tracking modality.

**TABLE 2:** Dosimetric comparison between plan types for the dose to the GTV, to the rib and to ipsilateral lung for the Synchrony and x‐sight spine tracking methods.

		GTV			Rib		Lung		
		# Scenarios V55Gy > 95% [%]	V55Gy [%]	*D*_max_ [Gy]	# Scenarios *D*_max_ < constraint [%]	*D*_max_ [Gy]	V20Gy [%]	V5Gy [%]	MLD [Gy]
Synchrony	GTV‐based	99.5	98.9 [98.6,99.3]	68.0 [67.5,68.5]	68	50.7 [43.8,57.6]	3.2 [2.4, 4.1]	18.0 [14.1,21.9]	3.5 [2.8,4.1]
	PTV‐based	99.5	99.9 [99.8,100]	68.5 [68.2,68.8]	41	56.0 [50.2,61.8]	4.5 [3.2,5.7]	22.3 [18.4,26.2]	4.1 [3.3,5.0]
	DO PTV‐based	100	99.5 [99.3,99.8]	67.1 [66.3,67.8]	45	55.7 [50.7,60.6]	4.4 [3.2,5.5]	21.8 [16.2,27.5]	4.1 [3.2,5.0]
x‐sight spine	GTV‐based	100	99.7 [99.4,100]	68.1 [67.4,68.7]	91	49.0 [41.8,56.3]	6.9 [3.8,10.0]	30.2 [18.0,42.4]	5.5 [3.4,7.6]
	PTV‐based	99.9	100 [99.9,100]	70.0 [68.2,71.8]	55	54.7 [49.1,60.4]	9.3 [5.6,13.0]	35.0 [20.7,49.3]	6.8 [4.3,9.2]
	DO PTV‐based	100	100 [99.9,100]	68.1 [67.7,68.5]	55	54.5 [49.7,59.3]	9.0 [5.4,12.6]	34.6 [20.5,48.7]	6.6 [4.3,9.0]
	ITV‐based	99.8	99.7 [99.3,100]	69.8 [67.9,71.7]	85	50.2 [44.7,55.6]	7.6 [4.3,10.8]	31.5 [18.3,44.6]	5.9 [3.6,8.2]
	DO ITV‐based	99.8	99.7 [99.4,100]	67.9 [67.3,68.4]	86	49.1 [43.5,54.7]	7.3 [4.2,10.5]	31.5 [18.7,44.3]	5.9 [3.6,8.1]

Additionally, for cases where the rib dose constraint was relevant, we report the maximum dose to the rib, as well as the average number of scenarios where the maximum rib dose met the constraint.

For both tracking modalities, lung doses were similar between PTV‐ and DO PTV‐based plans, as well as between ITV‐ and DO ITV‐based plans. For the Synchrony tracking, robust optimization led to an average reduction in ipsilateral lung dose of 29% for V20Gy and 19% for V5Gy. The MLD was reduced by 0.6 Gy in average. For x‐sight spine tracking, robust optimization considering only positioning uncertainty (ITV‐based plan) reduced V20Gy by 18% and V5Gy by 10%. The MLD was reduced by 0.9 Gy in average. For robust optimization considering both positioning and organ motion uncertainties (GTV‐based plan), the averaged V20Gy was reduced by 26% and the V5Gy by 14%. The averaged MLD was reduced by 1.3 Gy. All *p*‐values for lung dose metrics were below the 0.05 significance threshold for both tracking modalities.

The maximum dose constraint on the GTV, set at 68.75 Gy during plan optimization, was respected for all plan types except the PTV‐ and ITV‐based plans of the x‐sight spine tracking modality, where the maximum doses reached 70.0 and 69.8 Gy, respectively. Using robust optimization, the number of scenarios in which the rib dose constraint was met increased, and the maximum dose on the rib decreased for both tracking modalities.

**FIGURE 1:**
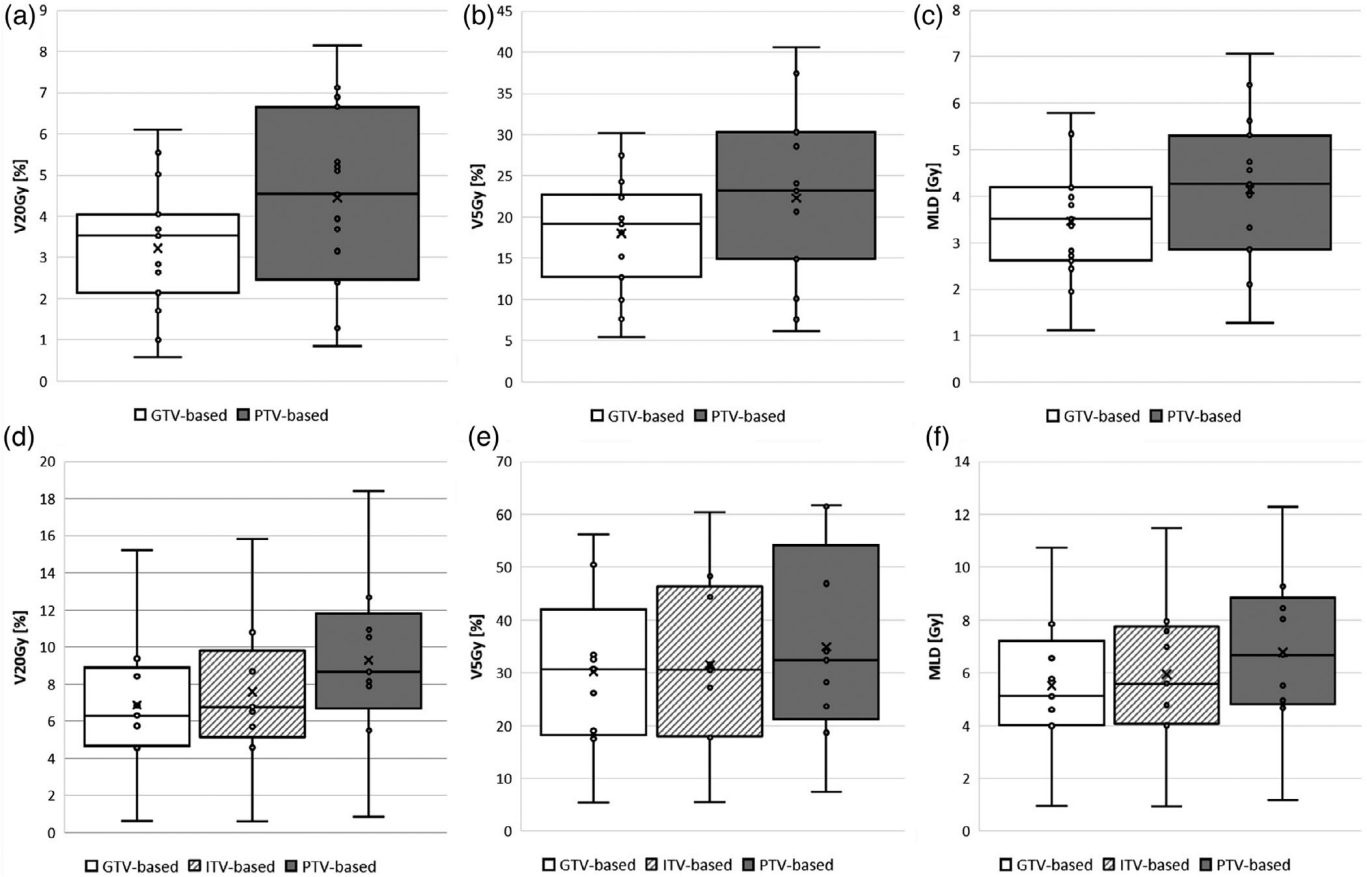
Dose to ipsilateral lung, (a) V20Gy, (b) V5Gy, and (c) MLD for the Synchrony tracking method and (d) V20Gy, (e) V5Gy, and (f) MLD for the x‐sight spine tracking method. The horizontal line in the boxes indicates the median value and the cross the mean. Results are shown for GTV‐, ITV‐ (for x‐sight spine tracking), and PTV‐based plans. Density override plans are not included, as their results were comparable to those of nonoverridden plans.

Figure 1 shows box plots of the V20Gy, V5Gy, and MLD received by the ipsilateral lung with Synchrony tracking (a, b, and c) and x‐sight spine tracking (d, e, and f) for the different plan types. Density override plans are not shown, as their results—as presented in Table 2—are comparable to those of the nonoverridden plans.

As shown in Figure 1, for all lung dose metrics and both tracking modalities, GTV‐based plans consistently have lower values for the first quartile (Q1), third quartile (Q3), interquartile range (IQR = Q3‐Q1), and median. ITV‐based plans (for x‐sight spine tracking), follow, with PTV‐based plans showing the highest values overall.

Table 3 presents the reduction in lung dose (ΔV20Gy, ΔV5Gy, and ΔMLD) between PTV‐based and GTV‐based plans for both tracking modalities. It also shows the correlation between the PTV‐to‐GTV volume ratio and GTV volume with these lung dose differences. The coefficient *r* corresponds to Spearman's rank correlation, and *p* denotes the associated *p*‐value.

**TABLE 3:** Reduction in lung dose (ΔV20Gy, ΔV5Gy, and ΔMLD) between PTV‐based and GTV‐based plans for both tracking modalities, and Spearman correlations between the PTV‐to‐GTV ratio and GTV volume with the corresponding lung dose reductions. *r* indicates the Spearman correlation coefficient, and *p* denotes the corresponding *p*‐value.

		PTV‐to‐GTV volume ratio		GTV volume	
Correlation		*r*	*p*	*r*	*p*
Synchrony	ΔV20Gy = −28.9%	−0.44	0.1	0.32	0.2
	ΔV5Gy = −19.3%	−0.35	0.2	0.12	0.7
	ΔMLD = −14.6%	−0.18	0.5	0.0	0.9
x‐sight spine	ΔV20Gy = −25.8%	0.12	0.8	0.10	0.8
	ΔV5Gy = −13.7%	−0.10	0.8	0.10	0.8
	ΔMLD = −19.1%	0.10	0.9	0.0	1.0

Table 4 shows the average number of MU and aperture size for each plan type in both the Synchrony and x‐sight spine tracking modalities. With Synchrony tracking, GTV‐based plans required an average of 38% more MU compared to the PTV‐based plans. With x‐sight spine tracking, GTV‐ and ITV‐based plans used on average 59% and 70% more MU, respectively, than the PTV‐based plans. The mean aperture size was reduced in robustly optimized plans compared to PTV‐based plans. As a result, the volume receiving 55 Gy on the planning CT decreased by an average of 25% using GTV‐based optimization compared to PTV‐based with Synchrony tracking. For x‐sight spine tracking, the reduction was 19% for ITV‐based and 35% for GTV‐based planning, both compared to PTV‐based plans.

**TABLE 4:** Average number of MU and aperture size for Synchrony and x‐sight spine tracking modalities.

	Synchrony			X‐sight spine				
	GTV‐based	PTV‐based	DO PTV‐based	GTV‐based	ITV‐based	DO ITV‐based	PTV‐based	DO PTV‐based
MU	3893	2829	2751	5357	5741	5408	3368	3259
Aperture size [cm^2^]	3.7	4.4	4.3	5.3	5.7	5.8	7.3	7.4

## 4 DISCUSSION

The CyberKnife installation enables lung SBRT to be performed using two tracking modalities: Synchrony tracking, which accounts for lesion motion due to breathing during treatment delivery, thus eliminating the need to consider respiratory motion during plan optimization; and x‐sight spine tracking, which does not track respiratory motion during treatment delivery and therefore requires the consideration of this motion during planning. Positioning and organ motion uncertainties can be addressed by defining volumes such as the ITV (for organ motion) and PTV (for positioning), but this leads to an increase in the irradiated volume, part of which corresponds to healthy lung tissue, and subsequently raises the probability of complications. Robust optimization, however, incorporates these uncertainties into the optimization process by defining error scenarios, reducing the irradiated volume of healthy tissue and hence minimizing the risk of complications.

For both tracking modalities, the average percentage of scenarios where GTV coverage exceeded 95%, along with the average GTV coverage, remained above 99% for all plan types. Thus, robust optimization, through GTV‐ and ITV‐based plans, did not compromise the dose to the tumor, presumably maintaining tumor control comparable to the PTV‐based plan. The density override of the PTV and the ITV to 1 g/cm^3^, along with GTV‐based planning, made it possible to control the maximum dose increase within the GTV compared to ITV‐ and PTV‐based plans. For ITV‐ and PTV‐based plans, the increase in maximum dose occurred because the GTV was irradiated with a fluence intended for low‐density tissue (i.e., the margin between the GTV and ITV and PTV). This benefit is more pronounced for the x‐sight spine plans where the volume of low‐density tissue was greater than in the Synchrony tracking modality, due to the use of an ITV.

Robust optimization led to a statistically significative reduction in lung dose. Although the lung dose was already relatively low in PTV‐based plans, any further decrease remains clinically beneficial—both in lowering the risk of radiation‐induced complications and in the context of an increasing incidence of re‐irradiation. Moreover, robust optimization resulted in a reduced interquartile range for all lung dose metrics for both tracking modalities. This decrease indicates a more homogeneous dose distribution among patients, suggesting improved treatment predictability, and reduced interpatient variability. No correlation was found between the reduction in lung dose metrics with either the PTV‐to‐GTV volume ratio or the GTV volume, indicating that neither the GTV‐to‐PTV margin nor the target volume significantly influences lung dose reduction. Specifically, in the case of x‐sight spine tracking, patients with large respiratory amplitude—reflected by higher PTV‐to‐GTV volume ratios—did not show an improved lung sparing. Overall, GTV volume was not a determining factor in lung dose reduction.

The use of the robust maximum dose objective on the rib has had a beneficial effect, with an increase in the number of scenarios for which the rib constraint was respected compared to PTV‐based optimization. However, even with robust optimization, the rib constraint was not respected in 100% of scenarios, probably due to the need to meet the minimal dose objective to the GTV. However, even in scenarios where the rib constraint was not fully respected, the maximum rib dose was still reduced by 5 to 6 Gy.

The superiority of robust optimization lies in its more realistic approach to managing uncertainties compared to conventional PTV‐based optimization. In PTV‐based planning, a static geometric margin is applied to account for all possible uncertainties, resulting in the irradiation of a volume that represents physically impossible uncertainties (e.g., a patient shift of 3 mm to the right and simultaneously 3 mm to the left). In contrast, robust optimization considers distinct and independent uncertainty scenarios, avoiding the irradiation of improbable configurations. This approach reduces dose to surrounding organs but requires increased MLC modulation. As a result, the average MLC apertures are smaller—since the irradiated volume per scenario is reduced—and the number of MUs is increased to ensure the prescribed dose to the GTV. This dose reduction to organs at risk, combined with an equivalent dose received at the GTV, reduces the risk of developing complications while maintaining a similar tumor control. In addition, GTV‐based planning removed the impact of PTV inhomogeneity, mostly seen on the maximum GTV dose.

The observed dose reduction in MLD and V20Gy for the ipsilateral lung in the ITV‐based plan with x‐sight spine tracking aligns with findings by Liang et al.^17^ in their study on ITV‐based robust optimization for VMAT planning in lung SBRT. However, we were unable to find any existing literature specifically reporting lung dose reductions for GTV‐based plans in this context.

One major limitation of using robust optimization was the significant increase in optimization time. In the case of Synchrony tracking modality, the optimization time was 3 to 4 times longer when using robust optimization compared to the PTV‐based planning approach. Similarly, for the x‐sight spine modality, the optimization time for an ITV‐based plan, which accounted only for positioning uncertainty, increased by a factor of 3 to 4. However, when considering a GTV‐based optimization, which accounts for both positional and organ motion uncertainties, the optimization time increased by a factor of 5 compared to ITV‐based and by a factor of 16 compared to PTV‐based plans. Moreover, verification of the robustness is recommended to ensure that the GTV receives the prescribed dose, further adding to the calculation time. These increased times must be considered when evaluating the practical application of robust optimization in clinical routines. To mitigate the increased optimization time associated with full robust optimization for x‐sight spine patients, a potential approach would be to reduce the number of 4D‐CT phases included in the robust settings. This could decrease the optimization time without necessarily compromising GTV coverage, as some respiratory phases may be similar. This approach will be investigated in a future study.

A limitation of this study is its focus solely on systematic uncertainties, affecting treatment plans in a predictable and consistent manner. However, random uncertainties, which introduce variability from one fraction to another, may impact the optimization differently. In a future study, we plan to separate systematic and random uncertainties to better understand their individual effects on the optimization process. This distinction will allow us to refine the optimization strategy, improving the robustness of the treatment plans and potentially enhancing their accuracy and clinical effectiveness.

## 5 CONCLUSION

The use of robustness in SBRT treatments of the lung with CyberKnife's Synchrony and x‐sight spine tracking modalities made it possible to reduce the dose received by the ipsilateral lung and the rib, while maintaining an equivalent dose to the GTV, ensuring potentially similar tumor control as the traditional PTV‐based planning. However, the current time‐intensive nature of robust optimization presents a challenge in clinical settings, which may limit its widespread adoption for now.

## CONFLICT OF INTEREST STATEMENT

The authors declare no conflict of interest.

## Supporting information



Supporting Information
