# Supplementary material for: Comparing dosimetric performance of robust and planning target volume‐based optimization in photon lung stereotactic radiotherapy
Source: Med Phys. 2025 Dec 29;53(1):e70219. doi: 10.1002/mp.70219 (PMC12746056; doi:10.1002/mp.70219)
Supplement: Supplementary file 1 — Supporting Information [file MP-53-0-s001.docx]

**SUPPLEMENTARY MATERIAL**

Supplementary Table 1 summarized the dose objectives on the target volume and dose constraints on the organs at risk for lung SBRT delivered in 5 fractions.

| Volume | Dose objectives and constraints |
| --- | --- |
| Target | V55Gy $\geq$ 95% |
|  | Dmax $\leq$ 68.75 Gy |
| Spinal canal | D0.03cc $\leq$ 30 Gy |
| Spinal cord | D0.03cc $\leq$ 25 Gy |
| Brachial plexus | D0.03cc $\leq$ 32 Gy |
| Rib | D0.03cc $\leq$ 43 Gy |
| Lung | V5 $\leq$ 50% |
|  | V20 $\leq$ 10% |
| Trachea/Bronchus | V18 $\leq$ 4 cc |
|  | D0.03cc $\leq$ 38 Gy |
|  | V35 $\leq$ 0.5 cc |
| Heart | V32 $\leq$ 15 cc |
|  | V40 $\leq$ 1 cc |
|  | V29 $\leq$ 0.5 cc |
| Aorta | V47 $\leq$ 10 cc |
|  | D0.03cc $\leq$ 53 Gy |
|  | V53 $\leq$ 0.5 cc |
| Esophagus | V20 $\leq$ 10 cc |
|  | V27.5 $\leq$ 5 cc |
|  | V32 $\leq$ 0.5 cc |
| Thoracic wall | V32 $\leq$ 30 cc |

Supplementary Table 1. Dose objectives and constraints for 5 fractions lung SBRT.
